# Sertraline induced acute hepatocellular liver injury in patient with major depressive disorder: a case report

**DOI:** 10.3389/fpsyt.2024.1456455

**Published:** 2024-08-01

**Authors:** Lubova Renemane, Elmars Rancans

**Affiliations:** ^1^ Department of Psychiatry and Narcology, Riga Stradins University, Riga, Latvia; ^2^ Riga Centre of Psychiatry and Addiction Disorders, Riga, Latvia

**Keywords:** sertraline, acute liver injury, hepatocellular liver injury, drug-induced liver injury (DILI), hepatotoxicity, selective serotonin reuptake inhibitor (SSRI)

## Abstract

This case report describes a patient with major depressive disorder (MDD) who developed acute hepatocellular liver injury after being treated with sertraline, a selective serotonin reuptake inhibitor (SSRI). The diagnosis of MDD was made two years prior, and the patient had previously responded partially to escitalopram and cognitive-behavioral therapy (CBT). Upon switching to sertraline 50 mg daily, the patient presented with severe symptoms indicative of acute liver injury, including elevated liver enzymes, jaundice, and gastrointestinal distress. Following the discontinuation of sertraline, the patient’s liver function tests gradually normalized over a 90-day period, confirming the diagnosis of sertraline-induced hepatotoxicity. This case underscores the importance of continuous monitoring for potential liver injury in patients treated with sertraline. The findings contribute to the existing body of evidence on the hepatotoxic risks associated with SSRIs and highlight the need for personalized treatment strategies to mitigate adverse effects and enhance patient safety. Further research is needed to explore the long-term safety and efficacy of sertraline, particularly in vulnerable populations.

## 1 Introduction

Major Depressive Disorder (MDD) is a complex and multifactorial mental health condition affecting millions globally, with approximately 7.1% of adults in the USA experiencing major depressive episodes annually, highlighting the substantial public health burden and impacting healthcare systems and productivity on a large scale (1). In Europe, the prevalence of MDD varies across countries, with some studies reporting rates as high as 6.38% in certain populations (2). Treatment for MDD includes pharmacotherapy and psychotherapy, with international guidelines recommending selective serotonin reuptake inhibitors (SSRIs) or serotonin-norepinephrine reuptake inhibitors alongside cognitive-behavioral therapy (CBT) as the first-line treatment (3–6). Sertraline, an SSRI widely used in the treatment of MDD, works by inhibiting the reuptake of serotonin into the presynaptic neuron and has very weak effects on norepinephrine and dopamine neuronal uptake (7). The pharmacokinetics of sertraline, including its metabolism to the active metabolite N-desmethylsertraline, are crucial for its therapeutic efficacy and safety profile (8). Additionally, sertraline has been found to influence brain-derived neurotrophic factor, which play a role in neurogenesis and the overall health of neural circuits, further contributing to its antidepressant effects (9).

Sertraline is preferred for its efficacy, safety, and tolerability across diverse patient populations, including those with co-morbid conditions. It is frequently administered to elderly patients due to its advantageous characteristics, such as a low incidence of drug-drug interactions, minimal renal impairment issues, and a limited effect on QT interval (10–13). It is associated with potential side effects such as nausea, insomnia, sexual dysfunction, diarrhea, dizziness, dry mouth, and fatigue, which are typically mild to moderate and tend to diminish over time (14). Although generally considered safe, sertraline has been reported to cause hepatotoxicity in some patients (15–17). Drug-induced liver injury (DILI) from sertraline is a rare but serious adverse event that can lead to significant morbidity. Symptoms of drug-induced liver damage are highly variable, with some patients remaining asymptomatic. Sertraline therapy can cause transient, asymptomatic elevations in serum aminotransferase levels and has been linked to rare cases of clinically significant acute liver injury. Reports of acute hepatitis secondary to sertraline use have highlighted marked elevations in liver enzymes, with or without accompanying jaundice (17–23).

Friedrich et al. conducted an observational study over 20 years, involving 184,234 psychiatric inpatients treated with antidepressants across 80 psychiatric hospitals, revealing 149 cases of drug-induced liver injury (0.08%) (24). Among the SSRIs, sertraline had a DILI probability of 0.05%. This probability was higher compared to escitalopram (0.01%), citalopram (0.02%), and fluoxetine (0.02%), similar to fluvoxamine (0.05%), but lower than paroxetine (0.06%). The most common clinical symptoms associated with DILI included nausea, fatigue, loss of appetite, and abdominal pain.

This case report presents an instance of acute liver injury following sertraline administration, contributing to the body of evidence on its hepatotoxic potential. By documenting this case, we aim to emphasize the need for vigilance among clinicians regarding this rare but serious complication, facilitating early recognition and management of sertraline-induced hepatotoxicity.

## 2 Case presentation

A 36-year-old Caucasian female with a diagnosis of MDD had been receiving sertraline 50 mg for the past 40 days. Prior to the initiation of sertraline, her liver enzyme levels were normal. The patient was admitted to the general hospital with complaints of newly onset nausea, scleral jaundice, vomiting, dark urine, pale stool, epigastric pain, and a subfebrile temperature, all of which developed over the four days preceding her admission. She reported no history of alcohol or substance abuse, nor the use of antibiotics or herbal medications. Additionally, there was no history of diarrhea, recent travel, or risk factors for viral hepatitis. The patient was not pregnant and was utilizing barrier contraception with condoms.

### 2.1 Background history

The patient has no family history of liver disease, mental illness, or substance abuse disorder. Her early developmental milestones were achieved without any delay. She later pursued higher education, earning a degree in economics, and has worked part-time for the past two years. She resides with her husband and their three-year-old child.

Two years ago, she was diagnosed with MDD according to DSM-5 criteria (25). Initially, the patient responded well to a regimen of escitalopram 10 mg/day in combination with CBT, which she continued for seven months before discontinuing the medication.

**Figure 1:**
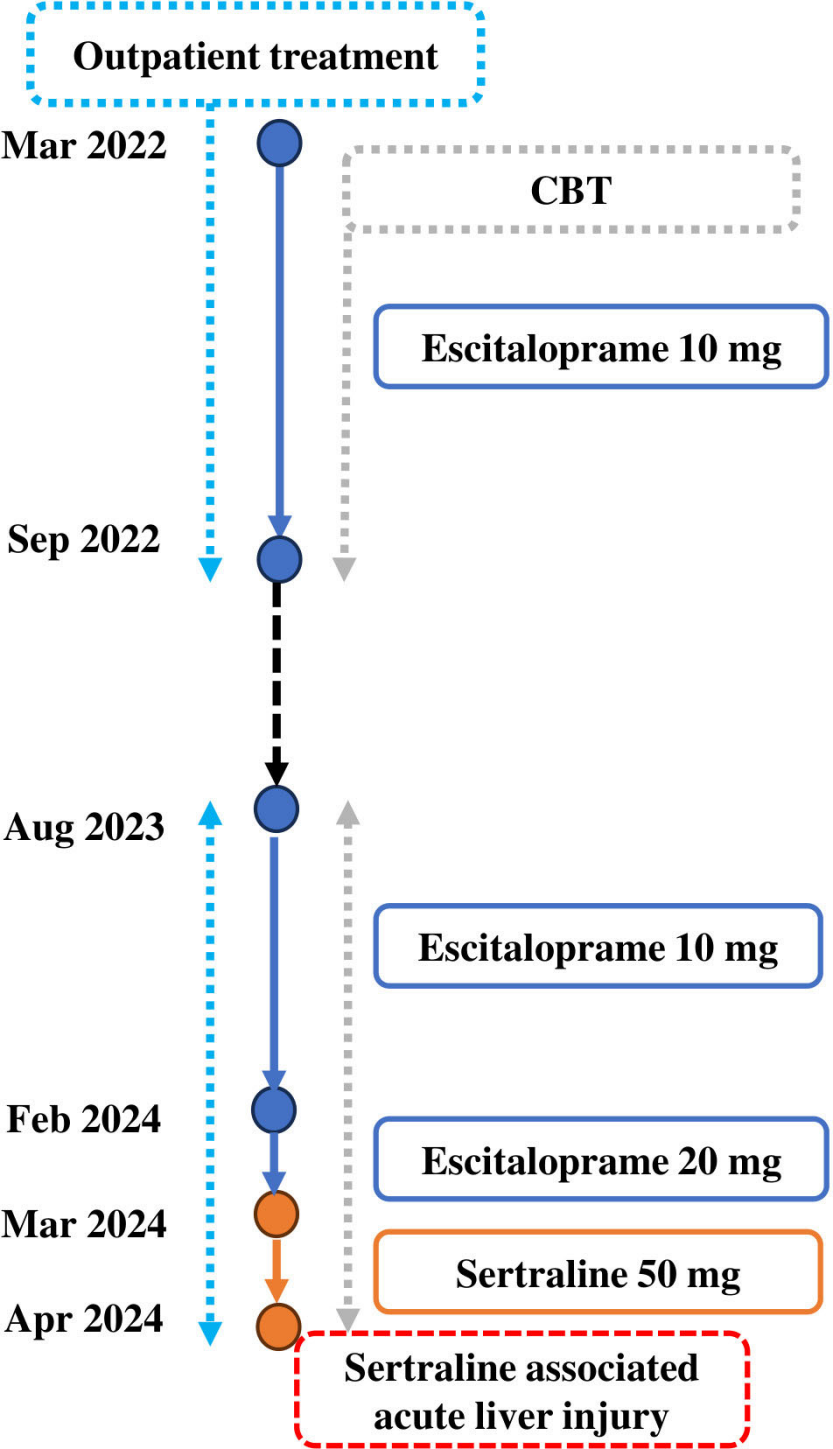
Timeline of outpatient treatment and onset of sertraline-associated acute liver injury.

One year later, she experienced a recurrence of depressive symptoms. After consulting with a psychiatrist, she was prescribed escitalopram 10 mg and referred to CBT. Despite five months of therapy, her symptoms worsened, prompting an increase in the escitalopram dose to 20 mg. However, she continued to report persistent symptoms of low interest, low energy, low mood, and decreased concentration. Consequently, her medication was switched to sertraline 50 mg daily (**Figure 1**).

### 2.2 Interventions and their outcomes

Upon admission to the hospital, sertraline was discontinued abruptly without tapering, and withdrawal symptoms were not observed. Liver function tests (LFTs) revealed significantly abnormal results indicative of acute liver injury, characterized by a predominant transaminitis. Alanine transaminase (ALT) was elevated at 553 U/L (reference range: 7–34 U/L), aspartate transaminase (AST) at 236 U/L (reference range: 10–35 U/L), alkaline phosphatase (ALP) at 153 U/L (reference range: 30–120 U/L), and gamma-glutamyl transpeptidase (GGT) at 117 U/L (reference range: 0–38 U/L). Total bilirubin was markedly elevated at 87 mmol/L (reference range: 1.1–19.0 mmol/L), with direct bilirubin also at 87 mmol/L (reference range: <5.0 mmol/L). These findings were consistent with acute liver injury. Other blood results, including prothrombin time, serum albumin, and international normalized ratio, were within normal ranges. A comprehensive blood screen was performed to further evaluate the patient’s condition.

An autoimmune screen for antinuclear antibodies, antismooth muscle antibodies, antineutrophil cytoplasmic antibodies, antimitochondrial antibodies, and antibodies against liver kidney microsomal type 1, Sp100, gp210, liver cytosol type 1, soluble liver antigen, and coeliac antibodies was negative. The patient’s immunoglobulin levels (IgG, IgA, IgM) were within normal ranges, indicating no recent acute infection or active immune response.

The patient tested negative for Human Immunodeficiency Virus (HIV) (anti-HIV1, anti-HIV2, p24 antigen), Epstein-Barr virus, cytomegalovirus, and toxoplasma. Tests for Hepatitis A, B, C, and E viruses were also negative. Additionally, iron studies, copper levels, 24-hour urinary copper, caeruloplasmin, and α-1-antitrypsin levels were all within normal limits.

An abdominal ultrasound revealed no abnormalities, indicating no signs of hepatic or biliary pathology. Similarly, Magnetic Resonance Hepatopancreatography showed no evidence of hepatic or pancreatic pathology. A liver biopsy demonstrated preserved hepatic architecture without significant inflammatory changes, but it displayed morphological features consistent with intrahepatic cholestasis, moderate lymphocytic and macrophage infiltration in numerous expanded portal areas, with occasional inflammatory infiltrate cells within the lobules. No liver fibrosis was observed.

Based on the initial assessment, a range of differential diagnoses was considered due to the non-specific symptoms at presentation. These included viral hepatitis, autoimmune hepatitis, and biliary pathologies. Additionally, hereditary liver disorders such as Wilson’s disease and haemochromatosis were evaluated. These potential diagnoses were ultimately excluded through detailed imaging and extensive biochemical investigations. The Hepatocellular R factor was calculated to be 14, indicating a predominance of hepatocellular injury. This high R factor supports the diagnosis of liver injury, specifically hepatocellular damage, likely induced by sertraline. Sertraline-induced acute liver injury was then considered as a diagnosis.

### 2.3 Follow up

**Figure 2:**
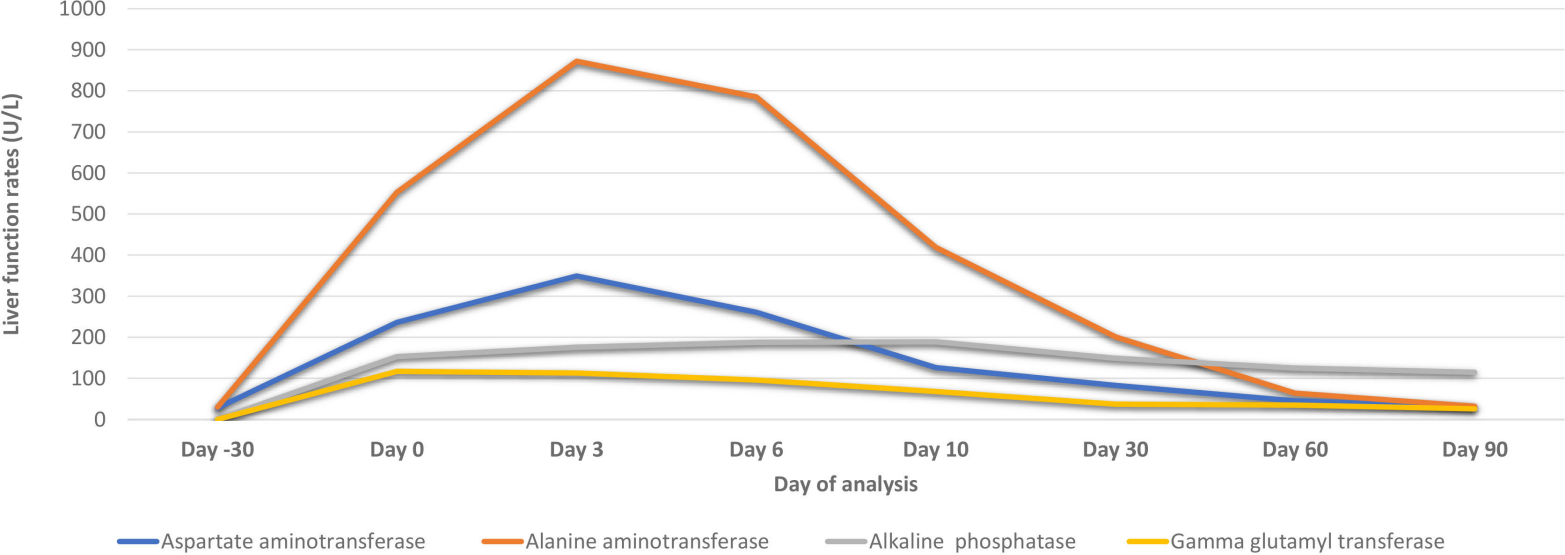
Biochemical parameters of the patient before and after hospital admission due to sertraline-induced liver injury.

**Figure 3:**
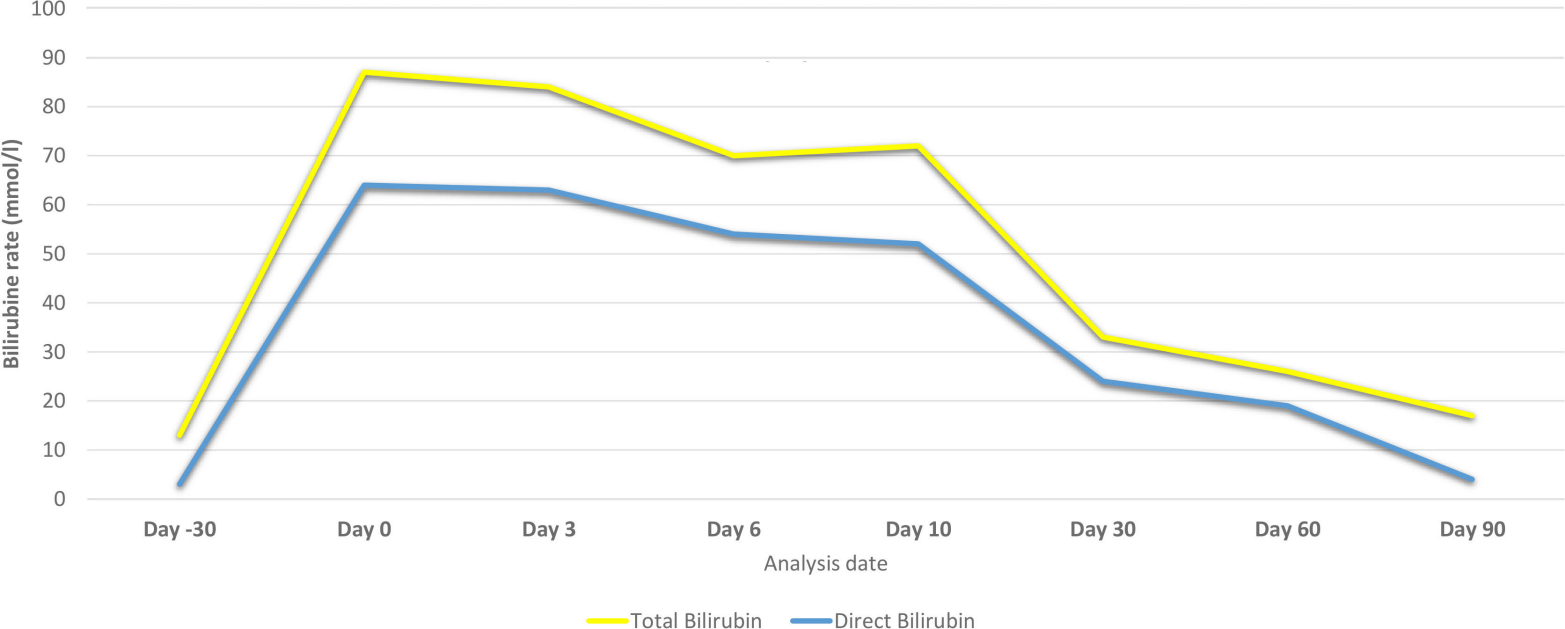
Bilirubin levels of the patient before and after hospital admission due to sertraline-induced liver injury.

The patient’s LFTs were monitored over a 90-day period to assess the extent and progression of liver injury, likely induced by sertraline. The changes in various biochemical markers are detailed in **Table 1**, **Figures 2**, **3**.

**Table 1:** Laboratory parameters of the patient before and after hospital admission due to sertraline-induced liver injury.

Date	AST (U/L)	ALT (U/L)	ALP (U/L)	GGT (U/L)	T. Bil. (mmol/L)	D. Bil. (mmol/L)
Day -30	28	30	n/d	n/d	13	3
Day 0	**236**	**553**	**153**	**117**	**87**	**64**
Day 3	**349**	**872**	**176**	**113**	**84**	**63**
Day 6	**261**	**785**	**188**	**96**	**70**	**54**
Day 10	**126**	**419**	**189**	**68**	**72**	**52**
Day 30	**83**	**201**	**149**	37	**33**	**23.8**
Day 60	**45**	**64**	**125**	35	**26**	**19**
Day 90	30	32	115	26	17	4

Day 0, admission to somatic hospital; AST, aspartate aminotransferase; ALT, alanine aminotransferase; ALP, alkaline phosphatase; GGT, gamma glutamyl transferase; T. Bil., total bilirubin; D. Bil., direct bilirubin; n/d, not done; numbers in bold reflect elevation out of the reference interval.

Over the first ten days, there was a significant initial elevation in liver enzymes and bilirubin levels, consistent with acute liver injury. Notably, there was a gradual improvement in these parameters by day 10, accompanied by an improvement in the clinical picture.

During the ongoing outpatient follow-up between days 30 and 90, the patient showed progressive and substantial improvement in liver enzyme levels, total and direct bilirubin, and albumin. This trend indicates a significant recovery from the initial acute liver injury, with liver function parameters approaching or reaching normal levels by day 90 after sertraline was discontinued.

Overall, the discontinuation of sertraline was followed by a marked improvement in liver function tests, supporting the diagnosis of sertraline-induced liver injury.

From day 30 onwards, the patient continued with CBT for the treatment of depression. No antidepressants were administered during this period as the patient declined pharmacotherapy. Future follow-ups are essential to understand further changes in symptoms and assess overall social functioning.

## 3 Discussion

This case illustrates severe hepatotoxicity with clinical presentations attributed to sertraline treatment for depression, evidenced by marked elevations in aminotransferase levels (AST and ALT), bilirubin, ALP, and GGT, which gradually normalized within 90 days following the discontinuation of sertraline.

This temporal relationship and normalization of liver function tests following the cessation of sertraline underscore the probable causative link between the drug and the liver injury. To further assess this relationship, we applied the Naranjo Adverse Drug Reaction Probability Scale, a structured tool designed to determine the likelihood that an adverse drug reaction is due to the drug rather than other factors (26). The Naranjo Scale, consisting of ten questions answered with “yes,” “no,” or “do not know,” categorizes the adverse drug reaction as definite, probable, possible, or doubtful. In our case, the application of the Naranjo Algorithm resulted in a score of 7 points, indicating a probable causal relationship between sertraline and the acute liver injury observed in the patient.

The hepatic pattern of damage linked to DILI can be categorized into three types: predominantly hepatocellular, predominantly cholestatic, or mixed (a combination of hepatocellular and cholestatic), with antidepressant-associated DILI generally more frequently presenting as hepatocellular (27). Our case aligns with previous reports that have shown sertraline-associated hepatocellular patterns of acute liver injury, characterized by elevated aminotransferase levels (AST and ALT) and other liver enzymes (18, 20, 28, 29).

Additionally, the suspected mechanisms of sertraline-associated DILI across available case reports include immunologic reactions, idiosyncratic reactions, a combination of immunologic and idiosyncratic reactions, and immuno-allergic mechanisms (29–32). The liver biopsy findings of moderate lymphocytic and macrophage infiltration in the portal areas, along with occasional intralobular inflammatory cells, indicate an ongoing inflammatory process in the liver in the present case. This is consistent with immune-mediated liver conditions associated with sertraline-induced liver injury.

Several studies have provided evidence of the favorable prognosis of DILI, with less severe forms resolving rapidly, although the normalization time for liver function in cases of acute liver injury varies widely across studies (21, 33). Our results are in agreement with previously reported cases, showing a resolution time of three months post-cessation of sertraline (20, 29). However, some studies have documented an aminotransferase normalization period of 5**–**6 months (30, 31, 34). Our patient exhibited a more rapid recovery, which may be attributable to the administration of sertraline as monotherapy at a low dose, the absence of concurrent pharmacotherapies, the absence of comorbid illnesses, younger age, and the lack of other medical risk factors.

The susceptibility of an individual to DILI is influenced by various genetic and epigenetic factors, as well as age, gender, body weight, alcohol consumption, preexisting liver illnesses, and comorbid medical illness (11, 19, 24, 35–37). For instance, a literature review noted that while low doses of sertraline (e.g., 50 mg) are less likely to cause significant liver injury, higher doses can lead to marked increases in aminotransferase levels (AST and ALT), bilirubin, and other markers of liver function (38). Some studies have suggested that antidepressant-induced liver injury is not dose-dependent and that age is not significantly related to the occurrence of DILI, aligning with our case (24). Nevertheless, our case and other published reports have documented instances of acute liver injury at standard therapeutic doses, emphasizing the importance of considering individual susceptibility factors, such as genetic enzyme polymorphisms affecting drug metabolism (35–37, 39).

Sertraline is metabolized primarily by the cytochrome P450 system, specifically CYP2D6 and CYP2B6 (40). Enzyme polymorphisms can impair the function of these enzymes, leading to increased drug levels and subsequent toxicity. For instance, a recent investigation explored the role of CYP-mediated metabolism in mitigating sertraline-induced toxicity using HepG2 human liver cancer cell lines expressing various CYP enzymes (41). The study suggested that CYP2D6, CYP2C19, CYP2B6, and CYP2C9 significantly reduce sertraline’s cytotoxicity through their metabolic activity. The authors concluded that DNA damage and topoisomerase inhibition are crucial mechanisms in sertraline-induced cytotoxicity, and that CYP-mediated metabolism plays a vital role in mitigating sertraline’s toxicity (17, 40). Moreover, several studies have indicated that the risk of acute liver injury increases when sertraline is co-administered with drugs metabolized by cytochrome P450 enzymes CYP2D6, CYP2C19, CYP2B6, and CYP2C9. These pharmacokinetic interactions can lead to altered plasma concentrations of these co-administered drugs (42–45). Furthermore, sertraline’s inhibition of these CYP enzymes can potentiate the adverse effects of concomitant medications through pharmacodynamic interactions (39). This mechanism results in elevated plasma levels of drugs metabolized by these enzymes, thereby increasing the risk of toxicity. For instance, Suen et al. reported a case of acute liver injury in a patient receiving sertraline combined with ranitidine, where the inhibition of CYP2D6 by sertraline led to increased ranitidine levels (20). Barahmania et al. described a case of liver injury attributed to the coadministration of sertraline and progesterone, suggesting that the mechanism could be explained by the combined use of both drugs (18). Furthermore, Mikhael et al. documented DILI secondary to the combination of sertraline and anabolic steroids, where the concurrent inhibition of CYP enzymes resulted in increased steroid levels, contributing to liver damage (46). These findings highlight the need for careful monitoring and potential dose adjustments when sertraline is prescribed alongside other medications metabolized by CYP enzymes, and it is crucial to consider the potential for both pharmacokinetic and pharmacodynamic interactions. In addition, collaborative care involving clinical pharmacists is highly beneficial for this patient population (47). Reviewing the literature, ethnic differences have been reported, suggesting variability in the occurrence of DILI across different populations. Our patient was Caucasian, and approximately 5 to 10% of Caucasians have reduced or non-existent CYP2D6 activity, placing them at increased risk of toxicity when receiving psychotropic treatments due to impaired drug metabolism (35, 36).

It is important to note that pharmacogenomic (PGx) testing may help identify patients at higher risk of adverse reactions, improving drug safety and efficacy. Gampa et al. reported on a patient case of cholestatic hepatitis after four weeks of sertraline 75 mg per day where a pharmacogenetic test revealed that the patient possessed specific genetic polymorphisms likely associated with increased drug levels and drug side effects due to poor metabolism of SSRIs (48). PGx testing can be considered prior to starting medications associated with DILI to avoid prescribing to those at higher risk for slower drug metabolizing capability.

In conclusion, this case highlights the rare but significant hepatotoxic potential of sertraline in the treatment of MDD. Despite the initial adverse effects, continuous monitoring and prompt discontinuation of the medication led to a substantial improvement in the patient’s liver function over a 90-day period. This underscores the importance of vigilance in managing sertraline therapy, especially considering individual susceptibility factors such as genetic polymorphisms and metabolic variability. Collaborative care involving clinical pharmacists is crucial to carefully monitor patients for any signs of liver injury, ensuring timely intervention. Future follow-ups are essential to understand further changes in symptoms, control tolerability, and assess overall social functioning. More evidence is needed regarding the long-term safety and effectiveness of sertraline, particularly in populations at higher risk for drug-induced liver injury.

## Data availability statement

The raw data supporting the conclusions of this article will be made available by the authors, without undue reservation.

## Ethics statement

Ethical approval was not required for the study involving humans in accordance with the local legislation and institutional requirements. Written informed consent to participate in this study was not required from the participants or the participants’ legal guardians/next of kin in accordance with the national legislation and the institutional requirements. Written informed consent was obtained from the individual(s) for the publication of any potentially identifiable images or data included in this article.

## Author contributions

LR: Conceptualization, Data curation, Formal analysis, Investigation, Resources, Visualization, Writing – original draft, Writing – review & editing. ER: Formal analysis, Project administration, Supervision, Writing – review & editing.
